# Neurological outcome after extracorporeal cardiopulmonary resuscitation for in-hospital cardiac arrest: a systematic review and meta-analysis

**DOI:** 10.1186/s13054-020-03201-0

**Published:** 2020-08-17

**Authors:** Benjamin Yaël Gravesteijn, Marc Schluep, Maksud Disli, Prakriti Garkhail, Dinis Dos Reis Miranda, Robert-Jan Stolker, Henrik Endeman, Sanne Elisabeth Hoeks

**Affiliations:** 1 Department of Anaesthesiology, Erasmus University Medical Centre, Rotterdam, The Netherlands; 2 Department of Public Health, Erasmus University Medical Centre, Rotterdam, The Netherlands; 3 Department of Intensive Care, OLVG, Amsterdam, The Netherlands; 4 Erasmus University Medical Centre School of Medicine, Rotterdam, The Netherlands; 5 Department of Intensive Care Medicine, Erasmus University Medical Centre, Rotterdam, The Netherlands

**Keywords:** In-hospital cardiac arrest, ECPR, Neurological outcome, Brain injury, CPC, Cerebral performance category

## Abstract

**Background:**

In-hospital cardiac arrest (IHCA) is a major adverse event with a high mortality rate if not treated appropriately. Extracorporeal cardiopulmonary resuscitation (ECPR), as adjunct to conventional cardiopulmonary resuscitation (CCPR), is a promising technique for IHCA treatment. Evidence pertaining to neurological outcomes after ECPR is still scarce.

**Methods:**

We performed a comprehensive systematic search of all studies up to December 20, 2019. Our primary outcome was neurological outcome after ECPR at any moment after hospital discharge, defined by the Cerebral Performance Category (CPC) score. A score of 1 or 2 was defined as favourable outcome. Our secondary outcome was post-discharge mortality. A fixed-effects meta-analysis was performed.

**Results:**

Our search yielded 1215 results, of which 19 studies were included in this systematic review. The average survival rate was 30% (95% CI 28–33%, *I*^2^ = 0%, *p* = 0.24). In the surviving patients, the pooled percentage of favourable neurological outcome was 84% (95% CI 80–88%, *I*^2^ = 24%, *p* = 0.90).

**Conclusion:**

ECPR as treatment for in-hospital cardiac arrest is associated with a large proportion of patients with good neurological outcome. The large proportion of favourable outcome could potentially be explained by the selection of patients for treatment using ECPR. Moreover, survival is higher than described in the conventional CPR literature. As indications for ECPR might extend to older or more fragile patient populations in the future, research should focus on increasing survival, while maintaining optimal neurological outcome.

## Introduction

In-hospital cardiac arrest (IHCA) is a serious adverse event in hospitalized patients that inevitably leads to death if not treated appropriately. It is associated with low survival rates at discharge and at 1-year follow-up (13%, 95% prediction interval 6–29%) [1, 2]. The use of extracorporeal membrane oxygenation (ECMO) in addition to chest compressions for cardiopulmonary resuscitation may improve survival after IHCA [3]. Recent guidelines state the use of ECMO for CPR (ECPR) as potentially beneficial for specific patient populations [4]; however, they also stress the lack of evidence for this novel technique [5]. To our knowledge, there is no large-scale evidence pertaining to neurologic outcomes after ECPR for IHCA [6, 7].

Survivors of cardiac arrest also suffer from neurological sequelae, which have been described as the post-cardiac arrest syndrome [8]. An important measure for neurological outcome is the aforementioned CPC. Although the CPC scoring suffers from limited discriminatory capacity and has a potential ceiling effect and possible overestimation of function, it is to date the most used outcome measure [9]. The neurological outcome of 1-year survivors after conventional CPR (CCPR) tends to be high: 92% of patients score a cerebral performance category (CPC) of 1 or 2 (95% prediction interval 82–97%) [2]. Another important neurological score is the Glasgow Outcome Scale (GOS). This outcome scale was developed for scoring outcome after acquired brain injury, but also is used to assess functional outcome after cardiac arrest [10, 11].

ECPR facilitates return of circulation, albeit artificial. However, it is much more uncertain whether this recovery of circulation translates into survival, or acceptable neurological outcome. Furthermore, the association between neurologic outcomes and prognostic factors should be elicited, in particular time to ECMO [12]. This systematic review aims to summarize the evidence on neurologic outcomes after hospital discharge of patients treated with ECPR for in-hospital cardiac arrest.

## Methods

### Literature search and selection criteria

This systematic review and meta-analysis is reported following the PRISMA and MOOSE guidelines for reporting of systematic reviews and meta-analyses of observational studies [13, 14]. For this systematic review, we performed a systematic search of all published data on post-discharge neurological outcome after IHCA treated by ECPR up to December 20, 2019. We used the search engines PubMed, EMBASE, Medline Ovid, Web of Science and Cochrane Central. Our searches contained the following keywords: in-hospital cardiac arrest, ECMO, neurological outcome, brain injury and neurological outcome. The exact search strategies are included in Additional file 1: Appendix 1*.*

Our inclusion criteria were as follows: (1) use of ECPR for in-hospital cardiac arrest, (2) adult patients, (3) reporting of neurological outcome (CPC or GOS), (4) clinical studies, and (5) written in English, German, French or Dutch. We included studies that reported outcome upon or after discharge from hospital. Studies were excluded if they did not fit inclusion criteria or if they were only published as abstract.

After the initial screening, the remaining articles were assessed by reading the full text. Studies often reported characteristics and outcomes of in-hospital and out-of-hospital cardiac arrest simultaneously. The authors of articles in which data for the IHCA cohort was not reported separately were contacted. Data extraction from selected studies was performed independently by two investigators (MD, PG) using a standardized form. Subsequently, the discrepancies were resolved by discussion with the other authors (BG, MS, SH).

### Definitions

The primary outcome was defined as favourable neurological outcome post-discharge from hospital using CPC or GOS score. A measurement was considered post-discharge, when the outcome was reported at discharge or later. For a description of the CPC and the GOS score, see Table 1 and Additional file 2: Appendix 2. A CPC score of 1 or 2 or a GOS score of 4 or 5 was defined as favourable outcome. The secondary outcome was post-discharge survival. If a study reported survival and neurological outcome at different follow-up moments, we ensured extracting the data for the same follow-up moment per study. Additionally, out of interest in the time to ECMO cannulation on the effect of ECPR, we extracted the average time to ECMO per study. Only the effect of the average time to ECMO cannulation on the primary outcome (favourable outcome) was investigated.

**Table 1:** Overview and characteristics of the included studies

Authors	Year and journal of publication	Study timeframe	Study type	Country	ECPR age median	Cardiac arrest to ECMO time (range)	Time of CPC assessment*
*Avalli* et al.	Resuscitation. 2012	Jan 2006 to Feb 2011	Retrospective	Italy	67 (61–73)	55 (40–70)	6 months
*Bednarcyzk* et al	Resuscitation. 2014	Feb 2008 to Sep 2013	Retrospective	Canada	–	49 ± 21	Discharge
*Blumenstein* et al.	Eur Heart J Acute Cardiovasc Care. 2016	Jan 2009 to Jan 2013	Retrospective	Germany	72 (55–72.9)	33.0 (19.0–47.0)	Discharge (30d)
*Chen* et al.	Lancet 2008	Jan 2004 to Dec 2006	Prospective	Taiwan	61.5 (18–74)	52.8 ± 37.2	Discharge
*Dennis* et al.	Int J Cardiol. 2017	2009–2016	Retrospective	Australia	–	40 (30–55)	Discharge
*Ellouze* et al.	Artificial Organs 2018	Jan 2011 to Jan 2015	Retrospective	France	–	60 (45–89)	6 months
*Fagnoul* et al.	Resuscitation. 2013	Jan 2012 to Jan 2013	Prospective	Belgium	–	55 (42–59.5)	Discharge from ICU
*Jung* et al.	Clin Res Cardiol. 2016	2002–2013	Retrospective	Germany	66 (56–78)	–	Discharge (30d)
*Lazzeri* et al.	Acute Cardiac Care 2013	Jan 2007 to Jan 2012	Prospective	Italy	54.8 ± 9 years (24–74)	51.9 ± 24.8	Discharge
*Lee* et al.	ann thorac surg. 2016	Jan 2004 to Dec 2013	Prospective	S. Korea	–	–	Discharge
*Lin* et al.	Resuscitation. 2010	2004–2006	Prospective	Taiwan	62.3 (21–73)	40 (16–150)	Discharge
*Liu* et al.	Interac cardiovasc thorac surg. 2011	Jan 2001 to Aug 2010	Prospective	Taiwan	53 (50–69)	–	Discharge
*Mazzeffi* et al.	J thorac cardiovasc surg. 2016	Jan 2010 to Dec 2015	Prospective	USA	57 ± 15 (34–86)	31 (15–52)	Discharge
*Peigh* et al.	J thorac cardiovasc surg. 2015	Jun 2010 to Jul 2014	Retrospective	USA	46 ± 12	54 ± 30	4-6w after discharge
*Pozzi* et al.	Ann thorac surg. 2019	Jan 2007 to Dec 2016	Prospective	France	46.2 ± 13.5 (18–76)	46.9 ± 19.0	Discharge
*Shin* et al.	Int J Cardiol. 2013	Jan 2003 to Jun 2009	Retrospective	S. Korea	59.9 ± 15.3	38.8	6 months
*Spangenberg* et al	Catheter Cardiovasc Interv. 2016	Jan 2014 to Oct 2015	Retrospective	Germany	–	42.9 ± 28.6	Discharge
*Stub* et al.	Resuscitation. 2015	Nov 2010 to Jul 2014	Prospective	Australia	–	56 (40–85)	Discharge
*Wang* et al.	Resuscitation. 2014	Jan 2007 to Aug 2012	Retrospective	Taiwan	55.7 ± 15.1	40 (15–162)	Discharge

*Neurological outcome and mortality was extracted at this follow-up moment

### Quality assessment

The quality of the included studies was evaluated using the method of Hayden et al. for prognosis studies in systematic reviews [15]. The quality assessment is based on six categories: (1) study population: whether the study correctly defines and describes the study population; (2) study attrition: whether the study was able to obtain a complete follow-up; (3) prognostic factor measurement: whether the study reports the most important prognostic characteristics; (4) outcome measurement: whether the neurological outcome was measured in a valid and robust way; (5) confounding measurement: whether the authors explored what influenced neurological outcome; and (5) account and analysis: whether the study reports a correct methodology of statistical analysis. Up to 2 points can be scored in each category. Therefore, the maximum score was 12 points, indicating high quality.

### Statistical analysis

For the analysis of the primary outcome, a fixed-effects model was used, because little heterogeneity was observed. Results of the meta-analyses are presented as pooled proportions with corresponding 95% confidence intervals (CI). Between-study heterogeneity was assessed using *I*^2^ statistic and the DerSimonian–Laird estimator for *τ*^2^. Moreover, heterogeneity was analysed by assessing statistical significance based on Cochran’s *Q* statistic.

Furthermore, because of specific interest in the relationship between time to ECMO and outcome in these patients, a meta-regression analysis was performed. A random intercept meta-regression analysis (binomial-normal model) was used with favourable outcome as outcome. This model is appropriate for meta-regression of probabilities, since it avoids the bias that occurs when a normal-normal model would be used for logit-transformed probabilities [16].

Finally, we considered multiple follow-up moments for our primary and secondary outcome. Therefore, a sensitivity analysis was performed for the studies that used the most frequently reported follow-up moment (i.e. at discharge).

All data was extracted into Microsoft Excel and then analysed in R (R Core Team (2013). R: A language and environment for statistical computing. R Foundation for Statistical Computing, Vienna, Austria). The packages used for the analysis were ‘meta’ and ‘metafor’, of which we used the ‘metaprop’, ‘forest’ and ‘rma.glmm functions’.

## Results

### Included articles

**Fig. 1:**
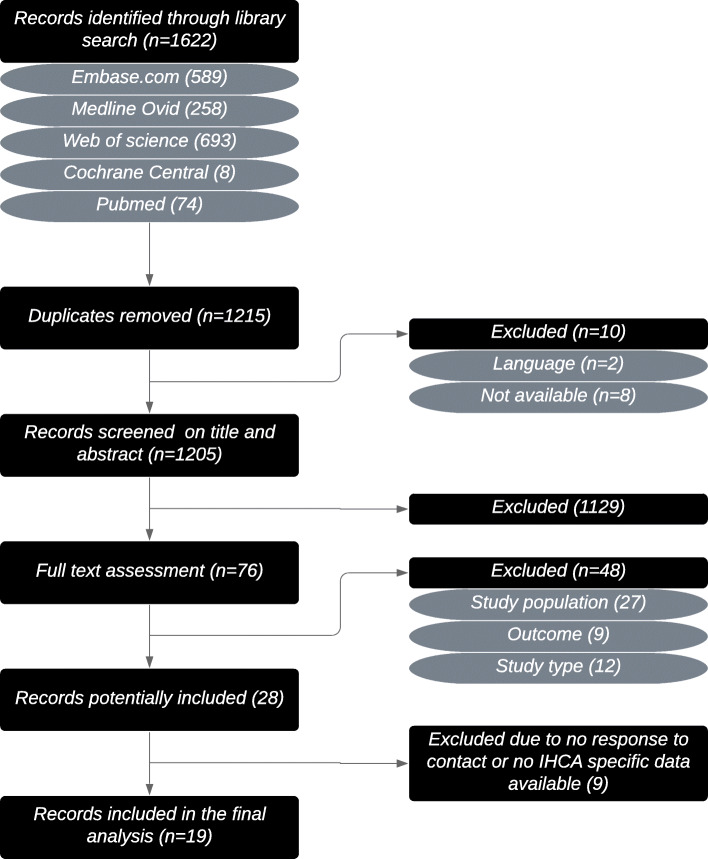
Flowchart showing the process of inclusion of studies. The search strategy was performed on 20 December 2019

Our search yielded 1215 results. Subsequently, 1130 articles were excluded by screening of title and abstract (2 because of a language different than Dutch, English, French or German). Full-text screening resulted in inclusion of 28 articles, of which 9 did not report characteristics and outcome of the IHCA cohort separately. For these articles, authors were contacted to provide this data for the IHCA cohort. None replied after multiple attempts; therefore, these studies were excluded. Finally, 19 articles were included [17–35] (Fig. 1).

The sample size ranged between 10 and 200 patients. The mean age ranged between 18 and 86. All studies were observational studies, of which 10 (53%) were retrospective (Table 1). All studies mentioned contra-indications. The most frequently reported contra-indications were CPR duration (58%), advanced age (58%), terminal cancer (84%), previous severe or irreversible brain damage (63%) and uncontrollable bleeding (63%). These are summarized in Table 2.

**Table 2:** Reported contra-indications for ECPR per study

Authors	CPR duration in minutes	Non-witnessed arrest	Severe comorbidity precluding ICU treatment	Advanced age (years)	Terminal cancer	Advanced CAD/heart failure	Non pre-selected patient categories*	Previous severe or irreversible brain damage	Liver cirrhosis	Renal failure	Uncontrollable sepsis	Uncontrollable bleeding/trauma	Irreversible (multi) organ failure	Arrest of septic origin	Coagulation disorder	BMI > 40	Weight < 30 kg	Aortic dissection	Extensive peripheral artery disease	Bedridden, care-dependant	At the discretion of the CPR team
*Avalli* et al.	< 30			> 75	x	X							x					x	x		
*Bednarcyzk* et al	< 15	x			x						x	x	x	x	x	x		x	x		
*Blumenstein* et al.		x	x		x	X		x				x			x			x			
*Chen* et al.	< 10	x		> 75	x		x	x				x									x
*Dennis* et al.																					x
*Ellouze* et al.	< 30	x		> 75	x			x				x									
*Fagnoul* et al.	< 10	x	x	> 65	x	X		x	x			x			x		x				
*Lazzeri* et al.	< 30			> 75	x			x				x	x	x							
*Liu* et al.	< 10				x		x	x				x									x
*Jung* et al.				> 74	x																x
*Lee* et al.					x																
*Lin* et al.				x	x	X		x				x									
*Mazzeffi* et al.	< 10						x														x
*Peigh* et al.				> 70	x	X		x			x	x									
*Pozzi* et al.	< 20	x			x	X		x				x									
*Shin* et al.				> 80	x			x				x	x	x							
*Spangenberg* et al.	< 20						x														
*Stub* et al.				> 65	x			x	x	x											X
*Wang* et al.	< 10	x	x	> 80	x			x				x	x							x	

*Some studies selected pre-specified groups based on cardiac arrest aetiology. Liu: acute myocardial infarction; Mazeffi: post-cardiac surgery; Chen/Spangenberg: cardiac origin; Stub: cardiac origin with ventricular fibrillation; Jung: cardiac origin or pulmonary embolism

Fifteen (79%) of the included studies had a score of ≥ 9 (out of 12) in the Hayden method for quality assessment (Table 3). Thirteen studies (68%) did not sufficiently adjust for confounding bias, while 18 studies (95%) reported important prognostic characteristics. Overall, high quality was observed for study participation (13 studies, 68%, received maximum scores), study attrition (14 studies, 74%, received maximum scores), outcome measurement (14 studies, 74%, received maximum scores) and analysis (17 studies, 89%, received maximum scores).

**Table 3:**
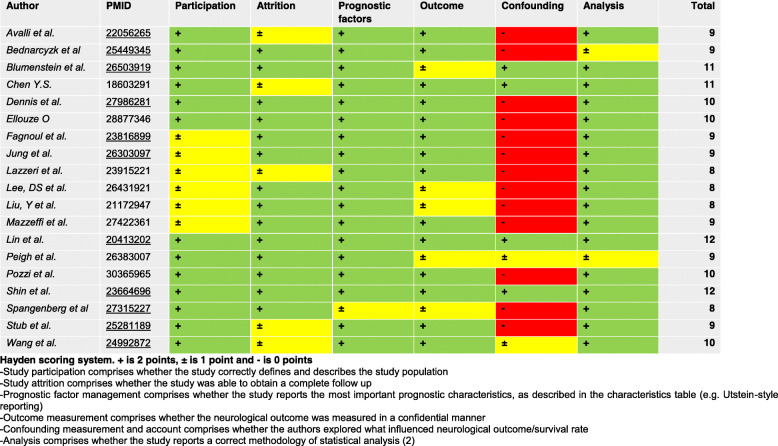
Risk of bias assessment, using the method of Hayden et al. for prognosis studies in systematic reviews

None of the included articles expressed neurological outcome in GOS. Six studies showed that all survivors were classified as CPC 1–2 [17, 18, 24, 26, 30, 34]. The largest study reported 52 patients with CPC 1–2 (84%) versus 10 patients with CPC 3–4 (16%) [20]. There was variation in the timing of assessment of outcome: 15 studies (79%) reported CPC and mortality at discharge, 2 (11%) studies reported CPC and mortality at 6 months, 1 (5%) study reported CPC and mortality at 4–6 weeks after discharge and 1 (5%) study reported CPC and mortality at discharge from ICU.

### Meta-analysis

**Fig. 2:**
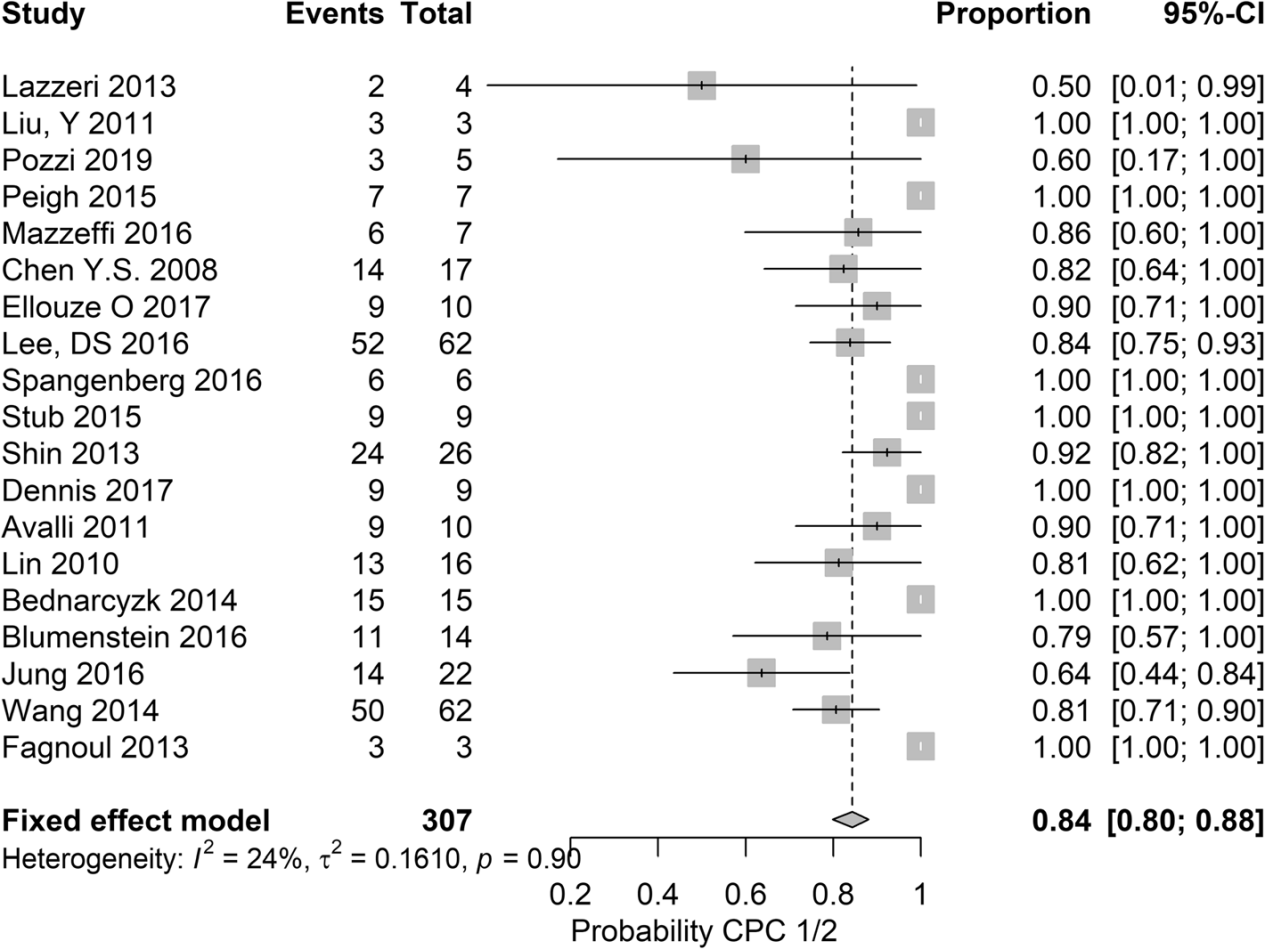
Forest plot showing the results for the primary outcome of this study, neurological outcome

**Fig. 3:**
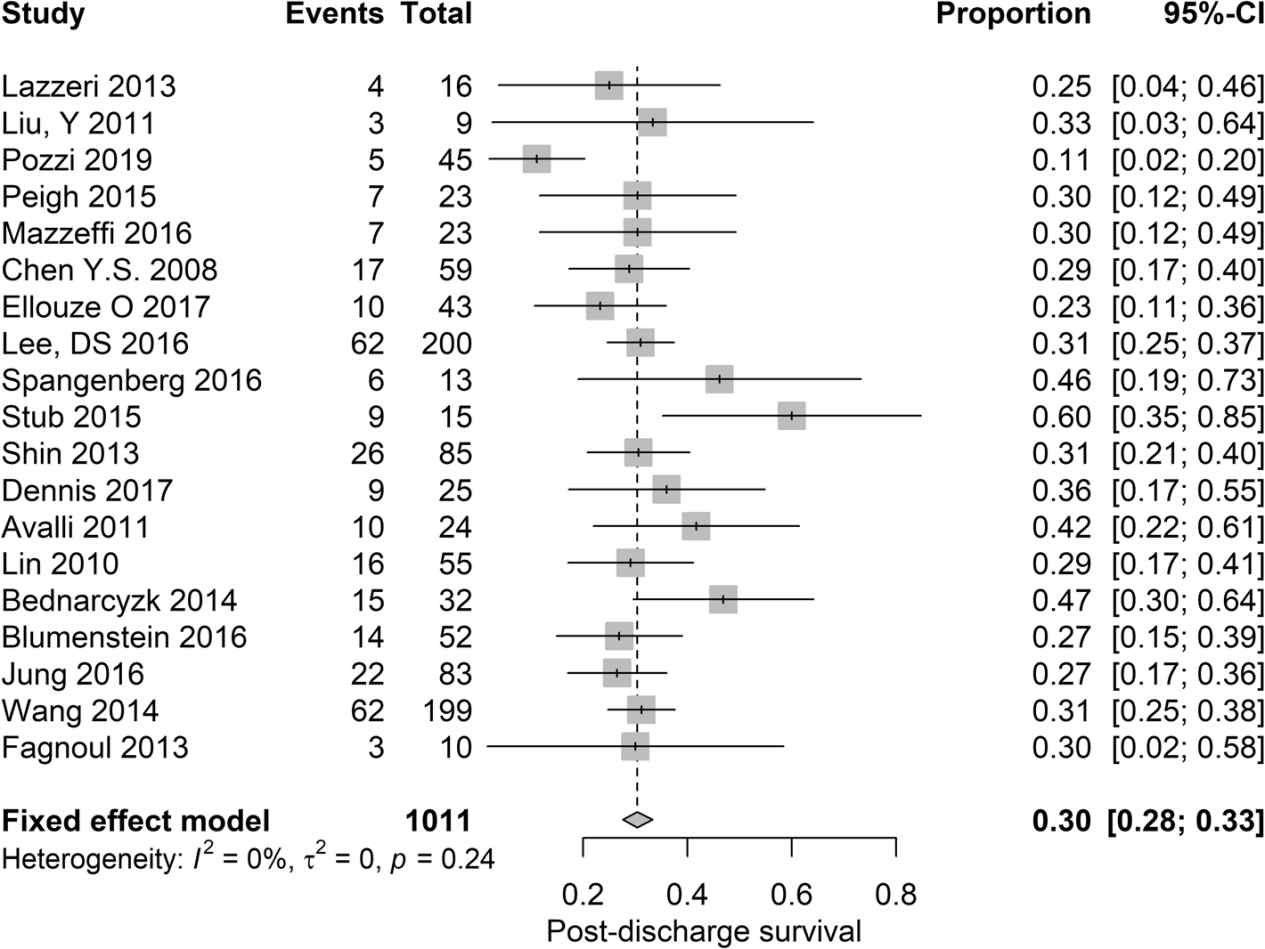
Forest plot showing the results for the secondary outcome of this study, post-discharge survival

The average post-discharge survival rate (i.e. discharge until 6 months) was 30% (95% CI 28–33%). Heterogeneity was low: *I*^2^ = 0%, *p* = 0.24. At the same follow-up moment in these survivors, the pooled proportion of favourable outcome was 84% (95% CI 80–88%). The heterogeneity was again low: *I*^2^ = 24%, *p* = 0.90 (Figs. 2 and 3).

As previously described, there was a variation in timing of assessment of outcome. In the 15 studies (79%) which reported survival to discharge, the pooled survival rate was 30% (95% CI 0.27–0.34%), with low heterogeneity (*I*^2^ = 0%, *p* = 0.15). In these survivors, the pooled proportion of favourable neurological outcome was 83% (95% CI 78–87%), with again low heterogeneity (*I*^2^ = 0%, *p* = 0.93).

#### Meta-regression

A total of 16 studies (84%) reported an average time to ECMO (time to cannulation/time to start ECMO), and the reported range was large (31–60 min). However, the OR per 10 min for favourable outcome was 1.29 (95% CI 0.73–2.29): favourable outcome was not explained by the average time to ECMO per study.

## Discussion

Our primary goal was to provide a comprehensive overview of current literature pertaining to neurological outcome after ECPR for in-hospital cardiac arrest. In post-discharge survivors, we found a high proportion of patients with a CPC 1–2 (84% [95% CI 80–88%]), which is lower than described for 1-year survivors CCPR (92% [95% PI 82–97%] [2]). Post-discharge survival was higher than reported for the general IHCA populations (30% [95% CI 28–33%] versus 17% [95% PI 13–23%] [2, 36]). We found little heterogeneity in outcome between studies.

Although neurological outcome is good, it remains inconclusive whether neurological outcome of patients receiving ECPR is better than patients receiving CCPR. We did find a lower percentage of “good” neurological outcome (CPC1–2) than in a systematic review in a conventional CPR population [2]. However, in this review, CPC score was a secondary outcome. In this review, the proportion outcome assessment was also specifically set for 1 year, rather than after hospital discharge. A systematic review aimed at comparing ECPR and CCPR suggests that the neurological outcome is better in IHCA patients treated with ECPR compared to CCPR [37]. Due to the observational nature of the studies included in these reviews, the selection of patients for ECPR could still lead to better outcomes for this group. For literature pertaining to OHCA, the same caveats apply [38, 39].

Comparing this study to the literature suggests that survival of IHCA patients undergoing ECPR is higher than IHCA populations who receive conventional CPR (chest compressions) [1, 2]. Our estimate of survival is also comparable the reported survival rate of adult ECPR patients by the ELSO registry [40]. This high survival might be explained by the selection of patients with a high chance of good outcome. The American Heart Association guidelines state that ECPR should be considered in patients for whom the suspected aetiology of the cardiac arrest is potentially reversible during a limited period of mechanical cardiorespiratory support [41]. In contrast, the European Resuscitation Council simply declares that the technique is a potential rescue therapy in patients where standard advanced life support (ALS) measures are not successful [5]. In practice, however, a much broader range of contra-indications are being used: this study found that the primary reported contra-indications were CPR duration, age, severe comorbidities such as terminal cancer or pre-existing neurological impairments and uncontrolled bleeding. These contra-indications are known to impact prognosis. Excluding these patients from ECPR effectively results in a higher survival compared to patients receiving conventional CCPR. Especially the age criteria are quite stringent and therefore likely affect the apparent survival [42], given the average age of the CPR population [43]. Moreover, the finding that we found substantially less heterogeneity in survival rates between studies than a systematic review of the CCPR literature [1, 2] also supports the hypothesis that this is a selected population. Nevertheless, part of the difference might be explained by the effect of ECPR versus CCPR on outcome [44–46].

On the other hand, ECPR is only indicated in patients with refractory cardiac arrest. Therefore, patients eligible for ECPR have, by indication, a worse prognosis than patients with conventional CPR as a portion of these patients ROSC after a short resuscitation period [47]. As a result, ECPR patients might not be the patient population with the most favourable outcome.

Evidence in the literature suggests that a longer time to ECMO time is associated with lower benefit of ECPR [48–51]. Bartos et al. suggest the association between time to ECMO and survival is explained by the metabolic derangements, which develop during prolonged low-flow time, leading to a worse outcome [52]. In our meta-analysis, this association between time to ECMO and survival is not found. However, most of the studies included in our meta-analysis do find a relationship between time to ECMO and survival, when this was investigated [18, 22–27, 34]. Possibly, our results can be explained due to an aggregation effect: our results imply that—because the variation in outcome between studies was small—differences in mean calculated time to ECMO do not explain differences in mean survival between studies. Additionally, our results might be explained by the long time to ECMO in the included studies (> 30 min). Given that the success rate of CPR is very low when the duration is longer than 30 min [53, 54], it might be more relevant to assess the effect of time to ECMO in when the time to ECMO is shorter. Since the effect of timing of ECPR on outcome impacts implementation, more high-quality evidence is needed.

Certain limitations should be taken into account. First, the time of CPC assessment was not the same for all studies. Most studies only scored CPC at the moment of discharge. This was not clearly defined in all studies. Some studies mentioned CPC scores at 6 months; others report a CPC score at discharge. We did show in a sensitivity analysis with the studies that reported data for the same follow-up moment that the estimates were very similar to the main analysis. However, a standardized and comprehensive assessment of neurologic and functional outcomes in cardiac arrest research is needed [9]. In spite of these differences, we encountered homogenous results, which suggest that the time of outcome assessment did not significantly influence the results: the neurological outcome and survival seem to remain constant at different follow-up times. Second, the included studies had two main shortcomings: they were relatively small (the largest study included 200 patients) and often reported their data non-standardized and non-structured, which complicated the process of data extraction. Remarkably, we observed little heterogeneity between these small studies, which enabled us to perform a fixed-effects meta-analysis. Finally, we were not able to do an individual patient data meta-analysis. Since heterogeneity between studies was found, the effect of prognostic factors on outcome in these patients could not be explored effectively. An individual patient data meta-analysis would enable this [55] and could be of interest for future research.

By showing that treating a selected group of IHCA patients with ECPR can result in a high proportion of good neurological outcome, this study illustrates what next step the field should take. When centres become more experienced, the indications of ECPR will shift towards a less selected, but probably also more fragile patient population: older patients with more comorbidities might be considered eligible for ECPR in the near future. Nevertheless, we should focus on treating these patients while maintaining such a high proportion of favourable neurological outcome.

## Conclusion

ECPR as treatment for in-hospital cardiac arrest is associated with a large proportion of patients with good neurological outcome (CPC 1–2). The large proportion of favourable outcome could potentially be explained by the selection of patients for treatment using ECPR. Nevertheless, both conventional and extracorporeal CPR are associated with low survival rates. The survival after ECPR, however, is higher than described in the conventional CPR literature. As indications for ECPR might extend to older or more fragile patient populations in the future, research should focus on increasing survival, while maintaining optimal neurological outcome.

## Supplementary information

**Additional file 1: Appendix 1.** Search strategy. Full search strategy, used in all databases.

**Additional file 2: Appendix 2.** Outcome measures. The definition of GOS and CPC scores, and the threshold for what is commonly defined as favourable outcome.

## Data Availability

Not applicable.
