# Supplementary material for: Neurological outcome after extracorporeal cardiopulmonary resuscitation for in-hospital cardiac arrest: a systematic review and meta-analysis
Source: Crit Care. 2020 Aug 17;24:505. doi: 10.1186/s13054-020-03201-0 (PMC7430015; doi:10.1186/s13054-020-03201-0)
Supplement: Supplementary file 1 — Additional file 1: Appendix 1. Search strategy. Full search strategy, used in all databases. [file 13054_2020_3201_MOESM1_ESM.pdf]

Appendix 1: Search terms

Pubmed 74

(in-hospital cardiac arrest[MeSH Terms] AND ecmo[MeSH Terms] AND (neurological outcome[Title/Abstract] OR brain injury[Title/Abstract] OR CPC[All fields] OR Cerebral Performance Category[All fields])).

embase.com 589

('heart arrest'/de OR 'cardiopulmonary arrest'/de OR 'sudden cardiac death'/de OR (((heart OR cardiac OR cardiopulmonary* OR circulat*) NEAR/3 (arrest OR death OR standstill)) OR asystol* OR SCD OR IHCA):ab,ti) AND ('Resuscitation'/exp OR (Resuscitation OR reanimation OR CPR OR (cardiac NEAR/3 'life support')):ab,ti) AND ('extracorporeal oxygenation'/de OR 'extracorporeal membrane oxygenation device'/de OR 'extracorporeal cardiopulmonary resuscitation'/de OR 'extracorporeal therapy'/de OR 'extracorporeal life support'/de OR (((extracorpor* OR extra-corpor*) NEAR/3 (oxygenat* OR life-support* OR resuscitation OR cpr)) OR ecmo OR ecls OR ecpr):ab,ti) AND ('hospital patient'/exp OR (hospitalized OR (hospital* NEAR/3 (patient* OR stay*)) OR inhospital* OR (in NEXT/1 (hospital OR patient)) OR inpatient OR IHCA):ab,ti) NOT ([animals]/lim NOT [humans]/lim)

Medline Ovid 258

("Heart Arrest"/ OR exp "Death, Sudden, Cardiac"/ OR (((heart OR cardiac OR cardiopulmonary* OR circulat*) ADJ3 (arrest OR death OR standstill)) OR asystol* OR SCD OR IHCA).ab,ti.) AND (exp "Resuscitation"/ OR (Resuscitation OR reanimation OR CPR OR (cardiac ADJ3 life support)).ab,ti.) AND (Extracorporeal Membrane Oxygenation/ OR (((extracorpor* OR extra-corpor*) ADJ3 (oxygenat* OR life-support* OR resuscitation OR cpr)) OR ecmo OR ecls OR ecpr).ab,ti.) AND ("Inpatients"/ OR (hospitalized OR (hospital* ADJ3 (patient* OR stay*)) OR inhospital* OR (in ADJ (hospital OR patient)) OR inpatient OR IHCA).ab,ti.) NOT (exp animals/ NOT humans/)

Web of science 693

TS=(((((heart OR cardiac OR cardiopulmonary* OR circulat*) NEAR/2 (arrest OR death OR standstill)) OR asystol* OR SCD OR IHCA)) AND ((Resuscitation OR reanimation OR CPR OR (cardiac NEAR/2 "life support"))) AND ((((extracorpor* OR extra-corpor*) NEAR/2 (oxygenat* OR life-support* OR resuscitation OR cpr)) OR ecmo OR ecls OR ecpr)) AND ((hospitalized OR (hospital* NEAR/2 (patient* ORstay*)) OR inhospital* OR (in NEAR/1 (hospital OR patient)) OR inpatient OR IHCA)))

Cochrane CENTRAL 16

((((heart OR cardiac OR cardiopulmonary* OR circulat*) NEAR/3 (arrest OR death OR standstill)) OR asystol* OR SCD OR IHCA):ab,ti) AND ((Resuscitation OR reanimation OR CPR OR (cardiac NEAR/3 'life support')):ab,ti) AND ((((extracorpor* OR extra-corpor*) NEAR/3 (oxygenat* OR life-support* OR resuscitation OR cpr)) OR ecmo OR ecls OR ecpr):ab,ti) AND ((hospitalized OR (hospital* NEAR/3 (patient* OR stay*)) OR inhospital* OR (in NEXT/1 (hospital OR patient)) OR inpatient OR IHCA):ab,ti)
