# Supplementary material for: Neurological outcome after extracorporeal cardiopulmonary resuscitation for in-hospital cardiac arrest: a systematic review and meta-analysis
Source: Crit Care. 2020 Aug 17;24:505. doi: 10.1186/s13054-020-03201-0 (PMC7430015; doi:10.1186/s13054-020-03201-0)
Supplement: Supplementary file 2 — Additional file 2: Appendix 2. Outcome measures. The definition of GOS and CPC scores, and the threshold for what is commonly defined as favourable outcome. [file 13054_2020_3201_MOESM2_ESM.pdf]

Appendix 2

Table 1: CPC scores

	CPC Grade	CPC Specification
Favourable neurological outcome	1	Full recovery or mild disability
	2	Moderate disability but independent in activities of daily living
Bad neurological outcome	3	Severe disability; dependent in activities of daily living
	4	Persistent vegetative state
	5	Dead

Table 2: GOS-scores

	GOS Grade	GOS specification
Bad neurological outcome	1	Dead
	2	Persistent vegetative state
	3	Severe disability; dependent in activities of daily living
Favourable neurological outcome	4	Moderate disability but independent in activites of daily living
	5	Full recovery or mild disability
